# Quantitative PCR assays to detect whales, rockfish, and common murre environmental DNA in marine water samples of the Northeastern Pacific

**DOI:** 10.1371/journal.pone.0242689

**Published:** 2020-12-02

**Authors:** Elizabeth A. Andruszkiewicz, Kevan M. Yamahara, Collin J. Closek, Alexandria B. Boehm

**Affiliations:** 1 Department of Civil and Environmental Engineering, Stanford University, Stanford, California, United States of America; 2 Monterey Bay Aquarium Research Institute, Moss Landing, California, United States of America; 3 Center for Ocean Solutions, Stanford University, Stanford, California, United States of America; University of Hyogo, JAPAN

## Abstract

Monitoring aquatic species by identification of environmental DNA (eDNA) is becoming more common. To obtain quantitative eDNA datasets for individual species, organism-specific quantitative PCR (qPCR) assays are required. Here, we present detailed methodology of qPCR assay design and testing, including *in silico*, *in vitro*, and *in vivo* testing, and comment on the challenges associated with assay design and performance. We use the presented methodology to design assays for three important marine organisms common in the California Current Ecosystem (CCE): humpback whale (*Megaptera novaeangliae*), shortbelly rockfish (*Sebastes jordani*), and common murre (*Uria aalge*). All three assays have excellent sensitivity and high efficiencies ranging from 92% to 99%. However, specificities of the assays varied from species-specific in the case of common murre, genus-specific for the shortbelly rockfish assay, and broadly whale-specific for the humpback whale assay, which cross-amplified with other two other whale species, including one in a different family. All assays detected their associated targets in complex environmental water samples.

## Introduction

Researchers across the globe have started monitoring aquatic species by identifying environmental DNA (eDNA) captured in water samples [1]. All organisms are constantly shedding DNA, which remains in an environment (i.e., soil, air, water), hence the term “environmental DNA,” or “eDNA” [1,2]. Monitoring by eDNA has several advantages over traditional monitoring methods such as trawl nets or visual identification. eDNA sampling is non-invasive and is independent of visually identifying the organism, thus the method is not subject to avoidance or identification error [3]. Small volume (i.e., 1 L) water samples can be analyzed for multiple taxonomic groups by applying molecular methods such as quantitative PCR (qPCR) or metabarcoding [4,5]. Major limitations of eDNA methods include uncertainty about the abundance or biomass present based on the eDNA concentration and the exact location and time of eDNA shedding from organisms [6–8]. However, the interest in using eDNA to identify marine organisms is growing and more assays are needed to identify important species.

Although results using eDNA metabarcoding provide information about many species from a single water sample, studies have demonstrated that the resulting data are not necessarily quantitative [9,10]. Furthermore, the metabarcoding method is prone to false negatives due to various technical challenges, including different target species having different affinities to PCR primers [7,9]. qPCR methods may be less prone to false negatives and studies have shown correlation between biomass and eDNA concentration [6,11,12]. The difficulty in using qPCR assays is that for every species of interest, a new assay must be designed and tested to evaluate its performance. The design of a qPCR assay depends upon having reliable genetic information from many individuals of the target species and finding a short region of a gene that will be specific to the target species and also suitable for the reaction chemistry of qPCR to ensure the assay is efficient. Finally, testing must be performed on other non-target species that are either co-occurring or closely related, as well as testing with water samples known to contain target DNA to evaluate performance in a complex matrix. Thus, the process can be laborious and time intensive. However, once the assay is developed, its use for biomonitoring has great potential.

We focus on three species of particular interest in the California Current Ecosystem (CCE): humpback whale (*Megaptera novaengliae*), shortbelly rockfish (*Sebastes jordani*), and common murre (*Uria aalge*). The CCE is a productive eastern boundary upwelling system along the west coast of the United States from Washington, USA to Baja California [13,14]. The CCE is well studied and particularly important to monitor due to its high primary productivity as a result of the upwelling system. The productivity in turn makes the CCE economically valuable. Therefore, it is necessary to monitor populations of taxa and biodiversity for conservation efforts. The CCE includes many protected areas, including federal National Marine Sanctuaries, state Marine Conservation Areas and Reserves, and local conservation areas.

Within the food web of the CCE, humpback whales, shortbelly rockfish, and common murre are three important organisms [15]. Humpback whales are protected under the federal Marine Mammal Protection Act enacted in 1972 when the species was close to extinction. Humpback whales are tertiary consumers and are frequently found in Monterey Bay due to the abundance of small fish and zooplankton [16,17]. Monitoring of humpback whale is traditionally carried out using visual surveys and can be sparse in space and time [18]. Recently, the use of citizens to conduct humpback whale surveys has gained momentum through citizen science campaigns [19].

Rockfish are secondary consumers in the CCE food web, consuming plankton and small fish [15]. The National Oceanic and Atmospheric Administration (NOAA) currently monitors populations of rockfish, including shortbelly rockfish, during its annual Rockfish Recruitment and Ecosystem Assessment Survey (RREAS) in the central CCE [15,20,21]. The RREAS has been conducted since 1983, and represents one of the longest time series of epipelagic juvenile fishes [22]. Shortbelly rockfish are one of over 100 species in the genus, *Sebastes*. Despite the long-term surveys, it can be difficult to make species assignments to juveniles that do not yet have identifying features of adults [23]. Net trawl surveys results in mortality of the captured organisms and can even result in mortality of individuals that escape the trawl net [24].

Common murre are piscivorous, top predator seabirds, that are abundant in the CCE. Their seasonal abundance has been correlated with availability of juvenile rockfish which can be a main source of prey [25,26]. Seabirds are monitored annually during the RREAS by visual survey (line transect), where an observer uses pre-determined distance intervals to estimate densities of seabirds [18,21]. This method is reliant on human observation and thus is subject to misidentification or false negatives [27].

The reliance on visual observations and trawling for abundance assessments of these three organisms gives rise to spatially and temporally sparse abundance data sets. The goal of this study is to develop eDNA assays to identify these organisms from water samples. We therefore designed and tested hydrolysis probe-based quantitative PCR (qPCR) assays targeting humpback whales (*Megaptera novaeangliae*), shortbelly rockfish (*Sebastes jordani*), and common murre (*Uria aalge*). To our knowledge, there are no published qPCR assays for these organisms. The assays were designed and tested *in silico*, *in vitro* in the laboratory, and *in vivo* using field samples suspected to contain eDNA from the organisms. Future efforts to test ecological hypotheses or carry out biomonitoring of these three marine species can utilize these assays; however, testing ecological hypotheses is not the goal of the present work. Finally, we include discussion of the challenges associated with qPCR assay design and suggestions for addressing these challenges.

## Materials and methods

### qPCR assay design

Assays were designed for the three organisms using the Geneious software (version 11.1.5), which uses a modified version of Primer3 (Version 2.3.7) [28]. For each organism, all sequences from the BLAST nucleotide (nt) database obtained from tissue vouchers were downloaded for a target gene (search completed on 16 August 2018). For humpback whale, the assay targeted the d-loop control region of the mitogenome. We limited the downloaded sequences for humpback whale assay design to include only those sequences obtained from organisms from the North Pacific (n = 180, S1 Table, including those from Jackson et al. (2009) [28] and Baker et al. (2013) [29]). The common murre and shortbelly rockfish assays both target the mitochondrial cytochrome c oxidase subunit I (COI) gene. These genes were chosen based on the availability of sequences for target species in the nt database. There were 16 common murre COI gene sequences and 5 shortbelly rockfish gene sequences at the time of our search, so all were downloaded for assay design (S1 Table).

Downloaded sequences were aligned using the default parameters in Geneious for a MUSCLE alignment and a consensus sequence was developed for each target. For all three targets, the assessment showed pairwise percent identity >97%. Thus, a representative sequence was chosen for each target to use for primer design from the National Center for Biotechnology Information (NCBI) database (humpback whale: GenBank Accession GQ353077, shortbelly rockfish: JQ354411, common murre: GU572157) (S1 Table). The “Design new primers” tool in Geneious was used in conjunction with the criteria outlined in Table 1, which included setting limits on product size, primer/probe size, primer/probe melting temperature (T_m_), and primer/probe GC content [30–33]. Ten primer/probe sets were returned by the software and were evaluated manually using the criteria outlined in Table 1, including optimizing the ΔT_m_ between primers and probe, no continuous occurrence of 4 or more of the same base, avoiding a thymine base at the 3’ end of the primer, including guanine or cytosine bases at the end of the primers, and no guanine at the 5’ end of the probe to reduce self-quenching of the fluorophore.

**Table 1:** Criteria used for designing primer/probe sets and criteria used to assess primer/probe sets *in silico*.

Criteria to design primers/probe	Criteria to select from designed primers/probes
• Product size between 100 and 200 bp	• Melting temperature between the primers less than 5°C
• Primer/probe size between 18 and 27 bp, optimal 20	• Melting temperature between the primers and probe of 8–10°C
• Primer melting temperature range 58–63°C, optimal 60	• No runs of 4 or more of the same base
• Probe melting temperature range 68–73°C, optimal 70	• Avoid a T base at the 3’ end of the primers
• GC content 35% - 65% for both primers and probe, optimal 50%	• C or G base at the 3’ end of the primers (to increase specificity)
	• 3 C and/or G bases in the last 5 base pairs at the 3’ end of the primers (to increase specificity)
	• No G base at the 5’ end of the probe (to avoid quenching of the fluorophore)
	• GC content of probe > 50%

The primer and probe set for each assay that adhered to the greatest number of criteria were subsequently used to test specificity *in silico* and sensitivity and specificity *in vitro*. These primer and probe sets are hereafter referred to as the “preliminary primer/probe sets”.

### *In silico* specificity testing

*In silico* testing was performed using the Geneious software “Blast” tool for the preliminary primer/probe sets using the default parameters. The primer/probe sequences were defined as the “Query” and the search was performed using the entire nucleotide collection (nt/nr) with the default parameters, except that the “maximum hits” criterion was set to 9,000. For each assay, database hits obtained for the forward primer, probe, and reverse primer were combined to identify entries in the nt database that contained sequences that matched both primers and probe sequences (search completed on 30 March 2019, S2 Table).

We generated sequence logo plots to illustrate the *in silico* specificity of the two primers and probe for each assay. Sequence logo plots were generated using the R package “ggseqlogo” [34] to illustrate the conserved and non-conserved nucleotide bases in the primer and probe binding regions [35]. Plots show (1) the relative frequency of base pair occurrence of the primers and probe by aligning all of the sequences for target species for each assay and (2) the relative frequency of base pair occurrence of the primers and probes using an alignment of non-target taxa. The latter was generated by aligning the regions of the forward/reverse primers and probe from all other non-target taxa within the same family of the target individual (e.g., all species within the family Balaenopteridae for humpback whale).

### Library of target and non-target samples for *in vitro* testing

To test and optimize each assay, we obtained tissue samples of individuals from both target species and non-target species to determine the sensitivity and specificity of each assay, respectively. We obtained tissue samples from four humpback whales, three shortbelly rockfish, and three common murre individuals (S4 Table). The humpback individuals were skin biopsies provided from either NOAA SWFSC (Permit # SR395) or The Marine Mammal Center (TMMC) (Permit # 19091). The shortbelly rockfish individuals were from fin clips or tissue provided by NOAA Southwest Fisheries Science Center (SWFSC) (no permit required). The murre individuals were provided by the California Department of Fish and Wildlife in Santa Cruz, California, USA in the form of liver tissue samples (no permit required).

Non-target species samples were obtained as tissue or archived tissue DNA extracts for *in vitro* specificity testing (S4 Table). The humpback whale assay was tested against a total of ten other species (n = 3 for 8 species, n = 1 for 2 species), including five other whale species (blue whale, fin whale, minke whale, grey whale, bowhead whale). The shortbelly rockfish assay was tested against 23 other species (n = 3 for 7 species, n = 1 for 16 species), including 12 different species within the rockfish genus, *Sebastes*. The common murre assay was tested against DNA from seven other birds (n = 1 individual for each of 5 species, n = 2 individuals for each of 2 species, S4 Table). S4 Table provides metadata on the samples used for specificity testing and permit information for marine mammals.

For non-target samples that were provided as tissue, DNA was extracted in our laboratory from each sample either using Qiagen DNeasy Blood and Tissue Kit (Qiagen, Valencia, CA) or by salting out [36] and the DNA extract was quantified using Qubit dsDNA HS assay (Life Technologies, Grand Island, NY). Samples that were provided as DNA extract were quantified using Qubit dsDNA HS assay (Life Technologies, Grand Island, NY).

### Assay optimization

Using one individual of each target species from our library, assays were optimized by varying primer/probe concentrations and annealing temperatures to achieve sensitive and high efficiency assays. For each assay, two non-target samples were included to check for specificity during assay optimization. Primer and probe concentrations tested ranged from 0.2–0.8 μM for primers and 0.1–0.2 μM for probes for a total of 6 combinations of primer/probe concentrations (primer/probe: 0.2/0.1, 0.2/0.2, 0.5/0.1, 0.5/0.2, 0.8/0.1 μM). The following reaction chemistry was used for assay development: Taqman Universal Mastermix II (1x), forward and reverse primer (varying concentration), probe (varying concentration), 2 μL of genomic DNA (gDNA) extract, and molecular-biology-grade water (Sigma-Aldrich, St. Louis, MO).

Annealing temperatures tested ranged from 60–64°C in 1°C increments. The cycling parameters were the same for all of the assays and were as follows: were 95°C for 5 minutes followed by 40 cycles of 95°C for 15 s, the annealing temperature being tested for 30 s and 72°C for 30 s. The C_q_ (quantification cycle) threshold was set at either 0.01 (humpback whale) or 0.02 (murre and shortbelly rockfish). No template controls (NTCs) were added with each plate tested using molecular grade water in lieu of DNA extract. All experiments were performed using a StepOne Plus Real-Time PCR System (Applied Biosystems, Foster City, CA).

Assays were also optimized to improve specificity by adding bovine serum album (BSA) if needed. For each assay, specificity, sensitivity, and efficiency were evaluated to determine the optimal primer and probe concentrations, annealing temperature, and the addition of BSA. Metrics to determine final assays included maximizing efficiency and R^2^ values of the standard curve, maximizing positive detections for target samples, and minimizing negative detections for non-target samples. After assay optimization, the preliminary primer/probe sets were all considered to be the final primer/probe sets and their sensitivity and specificity were determined before applying the assays to environmental samples.

### *In vitro* sensitivity and specificity testing

For the *in vitro* sensitivity testing, each primer/probe set was tested against a dilution series using DNA extracted from all individuals of each target species as template (n = 4 humpback whales, n = 3 shortbelly rockfish, n = 3 common murres). The highest concentration was 200 pg gDNA per reaction and the lowest was 2 fg per reaction; 1:10 dilutions of the highest concentration was used for intermediate dilutions. The dilution series was tested using triplicate reactions.

The *in vitro* specificity testing was conducted using all non-target samples from the library by including triplicate reactions of each sample, where 1–2 ng of gDNA was added per reaction. This test was conducted using the final primer/probe sets and cycling parameters after optimization.

### Data analysis

We identified the limit of quantification (“LOQ”) for each assay as the lowest concentration of target DNA for which all three triplicates were consistently assigned a cycle quantification (C_q_) value. Samples assigned a C_q_ value lower than that of the LOQ were considered positive. Samples that were not assigned a C_q_ value were considered non-detects (“ND”) and considered negative. No sample amplified at a C_q_ value higher than the that of the LOQ so there was no need to consider how to categorize measurements in this range. It should be noted that the units of the LOQ and LOD share the units of our standards which are ng of gDNA per μL of extract. Future studies using these assays will need to determine their own LOQ and LOD.

Sensitivity was calculated as the number of positive target samples divided by the total number of target samples. It is the ratio of true positives to the sum of false negative and true positives. Specificity was calculated by dividing the number of true negatives by the sum of the false positives and true negatives.

### Application *in vivo*

*In vivo* testing was conducted to demonstrate that the assays could detect target DNA in complex water samples, rather than highly concentrated gDNA samples extracted from tissue samples. Environmental water samples for *in vivo* testing were collected from Monterey Bay, California, USA and the Monterey Bay Aquarium Diving Birds Exhibit. Samples for testing the humpback whale assay were collected from the surface of the water column using a 10 L 10% HCl-acid washed, autoclaved polypropylene carboy (Nalgene, Rochester, NY) aboard the *R/V Paragon*. The samples used to test the shortbelly rockfish assay were collected aboard the *R/V Reuben Lasker* at 40 m depth using a Niskin array during conductivity-temperature-depth (CTD) casts. One liter water samples were collected from the Diving Birds Exhibit at the Monterey Bay Aquarium for testing the common murre assay using a 10% HCl-acid washed bottle. Water samples collected from the *R/V Paragon* were filtered and preserved with the Environmental Sample Processor using 0.22 μm pore size 255 mm diameter durapore filters (Millipore, Burlington, MA) [37]. All other water samples were filtered through 0.22 μm pore size 47 mm diameter durapore filters using DNA-clean, sterilized vacuum filtration devices. Filters were stored at -80°C until DNA extraction (within 16 months). DNA was extracted from filters using a previously described Qiagen DNeasy Tissue Extraction with bead-beating [38]. Aliquots of DNA were stored at -80°C until used as template in qPCR reactions (within 11 months).

Amplification of the field samples by qPCR was performed using 20 μL reactions with 2 μL of template using the reaction chemistry and cycling parameters of the optimized assays. All environmental samples were diluted 1:5 to reduce the chance of PCR inhibition. Standard curves for each assay were generated using serial dilutions of DNA from one target individual in order to quantify DNA in the environmental samples. Six ten-fold or five-fold dilutions were run in triplicate in each qPCR plate starting with 200 pg gDNA per reaction and ending with 2 fg gDNA per reaction for ten-fold dilutions or 64 fg gDNA per reaction for five-fold dilutions. Standard curve data were used to create a regression of DNA concentration per reaction versus C_q_ to calculate concentrations of unknown samples.

## Results

### Humpback whale assay final design and performance

The humpback whale assay targets the d-loop control region of the mitogenome. The primers/probe sequences are: F 5’ GCCGCTCCATTAGATCACGA 3', R 5’ TGGCCCTGAAGTAAGAACCAG 3', P 5' FAM - TCGCACCGGGCCCATCAATCGT - BHQ 3' (Table 2). After optimizing primer/probe concentrations and annealing temperature, reactions for amplification are 20 μL in volume with the following: Taqman Universal Mastermix II (1x), 0.2 mg/mL bovine serum album (BSA), forward and reverse primer (0.2 μM), probe (0.1 μM), 2 μL of DNA extract, and molecular-biology-grade water (Sigma-Aldrich, St. Louis, MO). The cycling parameters were as follows: were 95°C for 5 minutes followed by 40 cycles of 95°C for 15 s, 60°C for 30 s and 72°C for 30 s.

**Table 2:** Primers and probes designed for target organisms. Target organism, primer and probe sequences, gene target, fragment size, final concentrations of primers and probe, slope, intercept, limit of quantification, and efficiency of assay.

Species name	Common name	Gene targeted	Accession number used to design assay	Primer/probe sequences	Tm (^o^C)	G/C (%)	Target length (nt)	Final concentration (μM)	Ta (^o^C)	Cq threshold
*Megaptera novaeangliae*	Humpback whale	d-loop control region (mtDNA)	GQ353077.1	287F: 5' GCCGCTCCATTAGATCACGA 3'	60	55	151	0.2	60	0.01
				362P: 5' FAM - TCGCACCGGGCCCATCAATCGT - BHQ 3'	69	63.6		0.2		
				437R: 5' TGGCCCTGAAGTAAGAACCAG 3'	59.6	52.4		0.1		
*Sebastes jordani*	Shortbelly rockfish	COI (mtDNA)	JQ354411	169F: 5' CAGGAGCATCAGTCGACCTG 3'	60.2	60	176	0.2	60	0.02
				294P: 5' FAM - ACACCCTTATTTGTGTGGGCCGTCCT - BHQ 3'	68.2	53.8		0.2		
				344R: 5' GAGAAGGAGAAGGACAGCGG 3'	59.8	60		0.1		
*Uria aalge*	Commonmurre	COI (mtDNA)	GU572157.1	9F: 5' TGGCGCATGAGCTGGTATAG 3'	60	55	130	0.2	64	0.02
				34P: 5' FAM - ACCGCCCTAAGCCTGCTCATCCGT - BHQ 3'	70	62.5		0.2		
				138R: 5' TATTACAAAGGCGTGGGCGG 3'	60.7	55		0.2		

**Fig 1:**
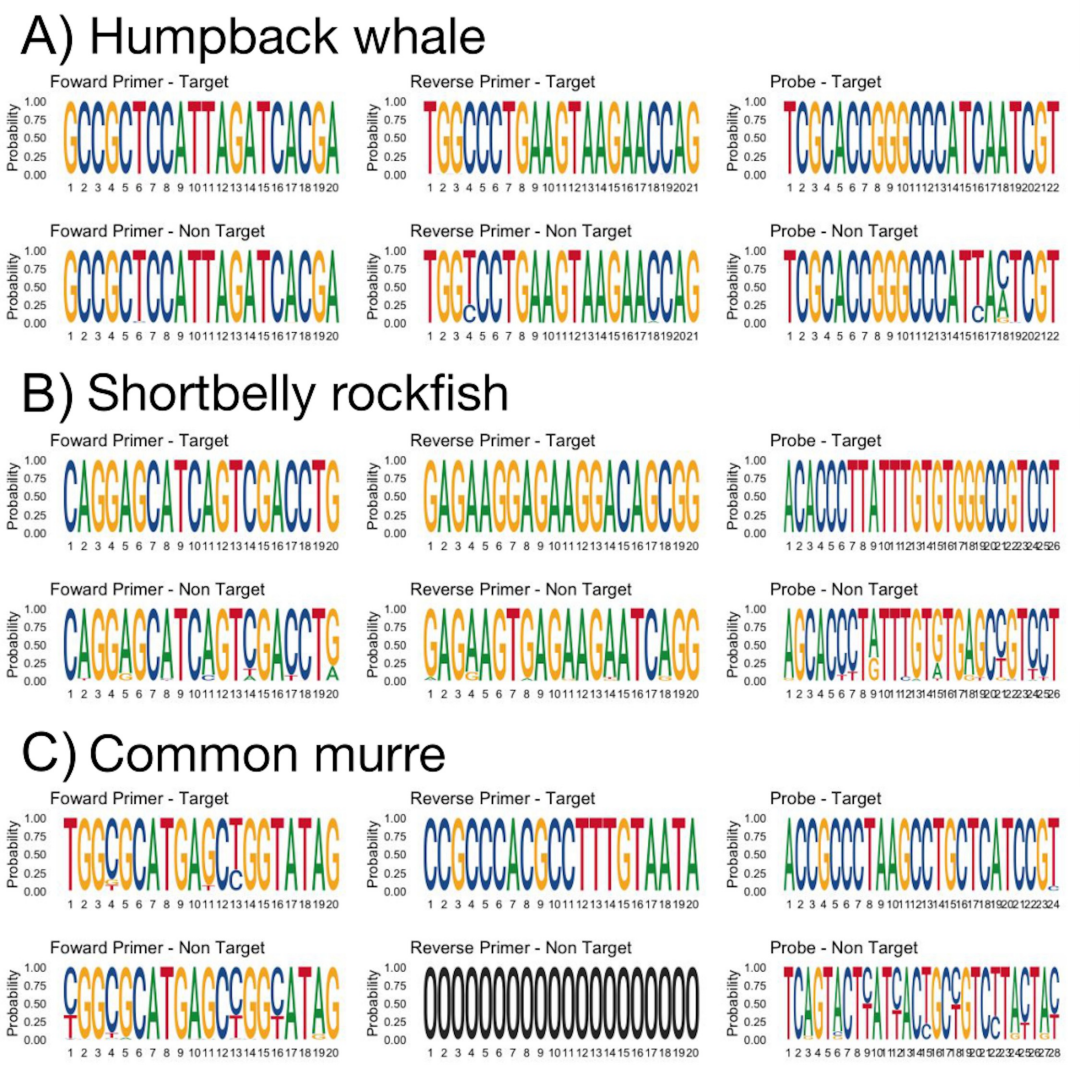
Sequence logo plots of *in silico* sensitivity and specificity of primers and probes for each qPCR assay. Panel A shows humpback whale, Panel B shows shortbelly rockfish, and Panel C shows common murre. For each panel, the top plots are generated by aligning the regions of the forward primer, reverse primer, and probe, respectively, of target species for each assay and the bottom plots are generated by aligning the same regions for non-target species. The x-axis is the order of base pairs in the primer or probe and the y-axis is the relative frequency of base pair occurrence, with the size of each letter corresponding to its relative frequency. Letters are stacked if multiple base pairs occur at that position in the primer or probe and relative frequencies always sum to 1. The reverse primer for common murre shows as “0” for all base pairs because no contigs were found when non-target species (within the family Alcidae) were aligned with the reverse primer sequence. All sequences are shown from 5’ to 3’.

During *in vitro* sensitivity testing, DNA from all four humpback whale individuals amplified, and thus the sensitivity of the assay was 100% (Table 3). These results match the *in silico* results demonstrated by the logo plot showing that target sequences have the exact same sequences in the binding region of the forward primer, reverse primer, and probe (i.e., all base pair relative frequencies are 1 at each position; Fig 1, Panel A, top plots).

**Table 3:** Sensitivity and specificity of each assay.

	Humpback whale	Shortbelly rockfish	Common murre
Total target individuals	4	3	3
True positives (TP)	4	3	3
False negatives (FN)	0	0	0
Total non-target individuals	26	37	9
True negatives (TN)	21	29	9
False positives (FP)	5	8	0
Sensitivity: TP/(FN+TP)	100%	100%	100%
Specificity: TN/(FP+TN)	81%	78%	100%

The specificity testing of the assay *in silico* returned matches to 4 additional species besides humpback whale: *Balaena mysticetus* (bowhead whale), *Balaenoptera acutorostrata* (minke whale), *Eubalaena australis* (Southern right whale), and *Eubalaena glacialis* (North Atlantic right whale) (S2 Table). *In vitro* testing included minke whale and bowhead whale (from the previous list), as well as blue whale, fin whale, and grey whale (Table 4), but did not include bowhead or Southern right whale. More generally, the *in silico* specificity illustrated in the logo plots indicated potential for cross-amplification from other taxa within the family, demonstrated by the relatively few differences in base pair frequencies in the assay between target and non-targets (1/20 in forward primer at low frequency, 2/21 in reverse primer at low frequency, and 2/22 in the probe; Fig 1, Panel A, bottom plots).

**Table 4:** Results of *in vivo* specificity testing. The three target organisms are shown on the right. Grey shading indicates that the assay was not tested for specificity using the organism on in the first column. “NA” indicates no amplification (i.e., assay is specific). For the non-target species that amplified, the C_q_ values are shown for each individual. 1–2 ng of gDNA was included in each reaction; see Fig 2 for C_q_ values for target individuals.

Specificity Testing			Target Organisms		
Species name	Common name	Individuals	*Megaptera novaeangliae*	*Sebastes jordani*	*Uria aalge*
*Uria lomvia*	Thick-billed murre	n = 1			NA
*Cepphus grille*	Black guillemot	n = 1			NA
*Alle alle*	Little auk	n = 2			NA
*Larus pacificus*	Pacific gull	n = 1			NA
*Larus delewarensis*	Ring-billed gull	n = 1			NA
*Larus heermanni*	Heermann's gull	n = 1			NA
*Sebastes auriculatus*	Brown rockfish	n = 1		NA	
*Sebastes carnatus*	Gopher rockfish	n = 1		NA	
*Sebastes caurinis*	Copper rockfish	n = 1		NA	
*Sebastes entomelas*	Widow rockfish	n = 3		NA	
*Sebastes flavidus*	Yellowtail rockfish	n = 3		NA	
*Sebastes goodei*	Chilipepper rockfish	n = 3		NA	
*Sebastes hopkinsi*	Squarespot rockfish	n = 3		24.8, 24.2, NA	
*Sebastes miniatus*	Vermillion rockfish	n = 1		NA	
*Sebastes nebulosus*	China rockfish	n = 1		NA	
*Sebastes paucispinis*	Bocaccio rockfish	n = 3		NA	
*Sebastes saxicola*	Stripetail rockfish	n = 3		25.6, 24.8, 24.5	
*Sebastes semicinctus*	Halfbanded rockfish	n = 3		23.7, 23.2, 22.9	
*Cottus spp*.	Sculpin	n = 1		NA	
*Ophiodon elongatus*	Lingcod	n = 1		NA	
*Sardinops sagax*	Pacific sardine	n = 1		NA	
*Scomber japonicus*	Pacific chub mackerel	n = 1		NA	
*Engraulis mordax*	Northern anchovy	n = 1		NA	
*Clupea pallasii*	Pacific herring	n = 1		NA	
*Seriola lalandi*	Yellowtail jack	n = 1		NA	
*Menidia menidia*	Silverside	n = 1		NA	
*Coryphaena hippurus*	Dolphinfish	n = 1		NA	
*Thunnus thynnus*	Bluefin tuna	n = 1		NA	
*Oncorhynchus kisutch*	Coho salmon	n = 1		NA	
*Balaenoptera musculus*	Blue whale	n = 3	NA		
*Balaenoptera physalus*	Fin whale	n = 3	NA		
*Balaenoptera acutorostrata*	Minke whale	n = 3	25.2, 22.7, 20.4		
*Eschrichtius robustus*	grey whale	n = 3	24.3, 36.7, NA		
*Balaena mysticetus*	Bowhead whale	n = 1	NA		
*Delphinus capensis*	Long-beaked dolphin	n = 3	NA		
*Delphinus delphis*	Short-beaked dolphin	n = 3	NA		
*Phoca vitulina*	Harbor seal	n = 3	NA		
*Zalophus californianus*	California sea lion	n = 3	NA		
*Carcharodon carcharias*	White shark	n = 1	NA		

**Fig 2:**
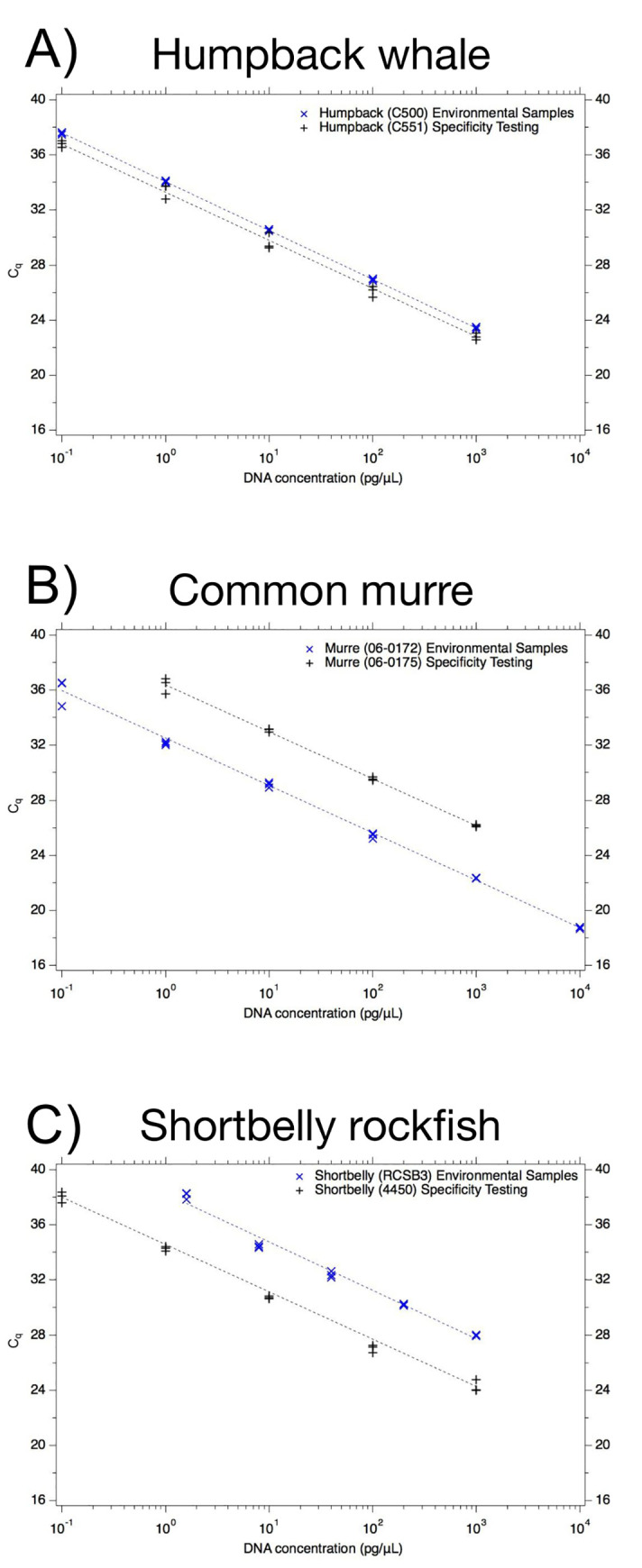
Performance of each assay. Cycle quantification threshold (C_q_) is shown on the y-axis and the x-axis is the concentration of DNA (pg gDNA/μL) in the reaction; 2 μL of template was added to each reaction for the dilution series. Panel A shows humpback whale (*Megaptera novaeangliae*), panel B shows common murre (*Uria aalge*), and panel C shows shortbelly rockfish (*Sebastes jordani*). For each plot, black crosses are the results of the individuals used for specificity testing (C551 for humpback whale, 06–0175 for common murre, 4450 for shortbelly rockfish). Blue x’s are the results of the individuals used for environmental sample testing (C500 for humpback whale, 06–0172 for common murre, RCSB3 for shortbelly rockfish). In the case of shortbelly rockfish (Panel C), some of the specificity testing was also performed using the standard curve used for environmental sample testing. Each concentration was run in triplicate reactions and in some cases, symbols are overlapping.

Although *in silico* testing suggested we might see cross-amplification between the humpback whale assay and bowhead whale during *in vitro* testing, the gDNA from bowhead whale (1–2 ng of gDNA per reaction) did not amplify. The gDNA from blue whale and fin whale also did not amplify. DNA from minke whale amplified (3/3 individuals) and grey whale also amplified (2/3 individuals) (Table 4, Fig 2). Given these results, the specificity of the assay was 81% (Table 3). The C_q_ values for minke and grey whale (1–2 ng of gDNA per reaction) were, on average, 22.7 and 30.5 (using just two reported C_q_ values). For comparison, the C_q_ for 2 μL of 1 ng/μL humpback whale gDNA is ~24 (Fig 2). The *in vitro* specificity testing included a standard curve constructed using extracted DNA from one individual (C551) and the efficiency was 92% with a LOQ of 0.1 pg/μL of gDNA extract (Fig 2, Panel A, black crosses).

For the humpback whale field samples, a standard curve was generated using extracted DNA from a different individual than that which was used for specificity testing (individual C500). The efficiency of the assay was 99% and the LOQ was 0.1 pg gDNA/μL of DNA extract (Fig 2, Panel A, blue x’s). All three qPCR triplicates of all three field samples were positive. After performing dimensional analysis from DNA mass per reaction to volume of water filtered, the average concentration of humpback whale DNA in the field samples was 0.06 pg/mL of water (Table 5).

**Table 5:** Results of environmental samples tested for each assay. **± is 95% confidence interval.** “BLOQ” represents below the limit of quantification. “ND” represents non-detect, meaning sample was not assigned a C_q_ value.

Assay target	Location of sample	Collection Method / Vessel	Concentration (pg/mL water)
Humpback whale	Monterey Bay, 36.797N, -121.847W	manual, *R/V Paragon*	0.06 ± 0.03
			BLOQ
			BLOQ
Shortbelly rockfish	Monterey Bay, 36.892 N, -122.462N	CTD, *R/V Reuben Lasker*	169.03 ± 27.55
	Monterey Bay, 36.914W, -122.407N	CTD, *R/V Reuben Lasker*	ND
Common murre	Monterey Bay Aquarium, Diving Bird Exhibit	manual	7.89 ± 0.46
			7.32 ± 1.04
			20.30 ± 2.06

### Shortbelly rockfish assay final design and performance

The shortbelly rockfish assay targets the COI gene. The primers/probe sequences were: F 5’ CAGGAGCATCAGTCGACCTG 3', R 5’ GAGAAGGAGAAGGACAGCGG 3', P 5' FAM—ACACCCTTATTTGTGTGGGCCGTCCT—BHQ 3' (Table 2). After optimizing primer/probe concentrations and annealing temperature, reactions for amplification were 20 μL reactions with the following: Taqman Universal Mastermix II (1x), forward and reverse primer (0.2 μM), probe (0.1 μM), 2 μL of DNA extract, and molecular-biology-grade water (Sigma-Aldrich, St. Louis, MO). The cycling parameters were as follows: were 95°C for 5 min followed by 40 cycles of 95°C for 15 s, 60°C for 30 s and 72°C for 30 s.

The *in vitro* sensitivity testing demonstrated that all individuals of shortbelly rockfish were positive and therefore assay sensitivity was 100% (Table 3). The logo plots support the sensitivity found *in vitro* as the forward primer, reverse primer, and probe all show unanimous consensus sequences when target sequences were aligned in the regions of the primers/probes (Fig 1, Panel C, top plots).

The assay was specific when tested *in silico* (S2 and S3 Tables), though the logo plots demonstrate that many of the non-target species within the family have similar sequences to the target sequences (Fig 1, Panel C, bottom plots versus top plots). The plots demonstrate that the probe adds the most specificity with more positions in the probe having different frequencies of base pair occurrence than the target (13/26 base pairs; Fig 1, Panel C, bottom plot). Despite the *in silico* specificity, the specificity testing *in vitro* found that DNA from 2 of 3 individuals of squarespot rockfish (*S*. *hopkinsi*), and 3 of 3 individuals of both stripetail rockfish (*S*. *saxicola*) and halfbanded rockfish (*S*. *semicinctus*) (Table 4) amplified, all of which also occur in the CCE. Thus, the specificity of the assay was 78% (Table 3). For *in vitro* specificity testing, non-target samples were compared to two standard curves; one was generated using one individual (4450) and had an efficiency of 94% and a LOQ of 0.1 pg gDNA/μL (Fig 2, Panel C, black crosses) and the other was generated using DNA extracted from tissue of one target individual of shortbelly rockfish (RCSB3) and had an efficiency of 93% and a LOQ of 1.6 pg gDNA/μL (Fig 2, Panel C, blue x’s). All non-target samples that were positive were tested using the RCSB3 standard curve. C_q_ values in the specificity testing of 1–2 ng of gDNA from these individuals ranged from 23–25 compared to a C_q_ of about 28 for 2 ng (2 μL of 1 ng/μL gDNA extract) of shortbelly rockfish.

Environmental samples were tested to quantify shortbelly rockfish DNA using the standard curve generated using RCSB3 (Fig 2, Panel C, blue x’s). DNA extracted from one of the two environmental water samples did not amplify (ND). The other environmental water sample had an average concentration of 169 pg/mL water (Table 5).

### Common murre assay final design and performance

The common murre assay targets the COI gene. The primers/probe sequences were: F 5’ TGGCGCATGAGCTGGTATAG 3’, R 5’ TATTACAAAGGCGTGGGCGG 3’, P 5’ FAM–ACCGCCCTAAGCCTGCTCATCCGT–BHQ 3’ (Table 2). After optimizing primer/probe concentrations and annealing temperature, reactions for amplification were 20 μL reactions with the following: Taqman Universal Mastermix II (1x), forward and reverse primer (0.2 μM), probe (0.2 μM), 2 μL of gDNA extract, and molecular-biology-grade water (Sigma-Aldrich, St. Louis, MO). The cycling parameters were as follows: were 95°C for 5 minutes followed by 40 cycles of 95°C for 15 s, 64°C for 30 s and 72°C for 30 s.

The *in vitro* sensitivity testing demonstrated that all target individuals were positive, and thus sensitivity was 100% (Tables 3 and 4). The logo plots demonstrate the assay sensitivity by the limited number of base pairs in the primers and probes with multiple frequencies at a specific site (3/20 in forward primer, 0/20 in reverse primer, 1/24 in the probe; Fig 1, Panel B, top plots).

The logo plots for the common murre assay demonstrate the assay specificity by showing the non-target alignment (Fig 1, Panel B, bottom plots) many different base pairs, particularly in the probe (7 of 24, or 30%, of base pairs having different frequencies than the target (Fig 1, Panel B, top plots). Furthermore, the region of the reverse primer was highly conserved and unable to be plotted because no contigs were found when comparing the non-target alignment to the reverse primer sequence (Fig 1, Panel B, bottom plot). The assay was specific both *in silico* (S2 Table, Fig 1) and *in vitro* (i.e., all non-target samples were ND) for the tissue tested (S4 Table, Table 5). Therefore, specificity of the assay is 100% (Table 3). The *in vitro* specificity testing included a standard curve constructed using extracted DNA from one individual (06–0175) and the efficiency was 97% with a LOQ of 1 pg/μL of gDNA extract (Fig 2, Panel B, black crosses).

The common murre assay was applied to samples from the Diving Birds Exhibit at the Monterey Bay Aquarium. A standard curve was constructed using a different individual than specificity testing (06–0172). The efficiency was 99% and the LOQ of 0.1 pg/μL of gDNA extract (Fig 2, Panel B, blue x’s). The average concentration was 11.8 pg/mL water in the three samples (Table 5).

## Discussion

We developed qPCR assays for three important organisms of the CCE: humpback whale, shortbelly rockfish, and common murre. The assays all had high sensitivity (100% for individuals tested), and the assays yielded positive detection of their targets in environmental samples or samples from an aquarium. However, specificity varied across assays with the common murre assay having high specificity (100%) but the humpback whale and shortbelly rockfish having cross-amplification. The humpback whale assay cross-amplified minke and grey whale both of which also are common in the CCE [18,39]. Thus, positives with the assay might indicate the presence of these other whale species. The shortbelly rockfish assay cross-amplified with three species of rockfish also known to be in the CCE. The rockfish genus *Sebastes* includes over 100 species and despite a wide geographical distribution, the phylogeny has been debated amongst researchers for years [23,40]. Therefore, the sub-optimal specificity is not particularly surprising for the rockfish assay. These specificity issues highlight the difficulties in designing qPCR assays, ranging from the availability of sequences in the public databases to biological challenges such as unclear phylogeny and cryptic species.

Design of specific qPCR assays relies upon correct and sufficient genetic data. Public databases such as NCBI’s GenBank contain many entries, however the accuracy and quality of data can be questionable. For example, a recent paper on scyphozoan jellyfishes revealed that the majority of database entries in GenBank for the species *Chyrsaora quinquecirra* are in fact *Chrysaora chesapeakei* [41]. The paper, published in 2017, confirmed the mis-identifications and incorrect entries by both morphological and genetic analyses, yet three years later none of the entries have been corrected in GenBank [41]. Furthermore, processing of Sanger sequencing data that is submitted to public databases requires visual assessment and careful processing and even one base pair difference in a target gene region can impact qPCR assay design and performance. Discrepancies or mistakes in the genetic data used for qPCR assay design can cause poor design and performance and misleading *in silico* specificity testing results. Public databases need better maintenance, a higher standard of metadata required for submission, and accountability for corrections if new evidence suggests that they were incorrect upon submission.

Assuming the genetic data used for assay design are correct, finding regions that are suitable for qPCR chemistry is another challenge for assay design. The targeted region must be relatively short (100–200 bp) in order to capture degraded fragments of eDNA left behind in the water column, but this criterion restricts the possible primer locations on the gene. The chemistry of the qPCR reaction requires certain ranges of melting temperatures, GC content, and other criteria such as the types of base pairs on the 3’ end of the primer in order for the assay to be successful and efficient [30–33]. Due to the narrow range based on the size restriction on the fragment, this can be a major challenge for qPCR assay design. Additionally, the probe, which is often around 25 bp long and thus can be up to 20% of the total target length, must be located in the region between the forward and reverse primers. A probe is included to increase specificity, but it is often difficult to identify a probe that would be successful given the assay’s chemistry requirements. Finally, in assay development there are several trade-offs to be made between assay performance. For example, raising the primer annealing temperature may increase specificity of the assay while simultaneously decreasing sensitivity. Depending on the assay application, these parameters can be optimized ideally with one set of primers and a probe, or many primer/probes can be tested *in vivo*. Furthermore, many studies that publish new qPCR assays provide insufficient detail about methodology, specifically regarding *in silico* and *in vivo* testing. Greater transparency in publications will help guide new efforts for qPCR assay design and testing.

Furthermore, there are differences in individuals from the same species that can produce different qPCR results. We used DNA extracted from tissue samples from different individual target organisms to create standard curves. For humpback whale, the tissue samples yielded similar standard curves when mass of DNA tested is plotted against C_q_ from the qPCR instrument. The common murre and shortbelly rockfish standard curves constructed using DNA from different individuals were different: the same input DNA mass yielded C_q_ values differing by approximately 4 C_q_. This suggests differences exist in mtDNA concentrations between the individual DNA extracts. For the two shortbelly rockfish tissue samples, one originated from a fin clip of an individual of unknown age and the other from tissue from the body of a juvenile individual. Both murre tissue samples originated from livers of two individuals of unknown age. The mass of DNA extracted from the various individuals represents contributions from nuclear DNA (nDNA) and mitochondrial DNA (mtDNA). The ratio of nDNA:mtDNA can vary between individuals of a specific species and among tissues in a single individual [42]. A study of their ratio in mice, for example, showed that the ratio varied based on the age (young versus old) of the mouse by a factor of 10 [43]. The ratio seems to be sensitive to DNA extraction method as well [44]. A recent study assessed the differences in eDNA degradation from water samples of a water flea (*Daphnia magna*) when quantified by targeting both a mitochondrial and nuclear gene region [45]. This study found that nuclear DNA was more abundant, however the lengths of the two target regions were also different and the authors acknowledge that other technical biases may be influencing the results such as primer affinity. Different nDNA:mtDNA ratios in the tissue extracts could explain the differences in C_q_ values seen between individuals in our study. This discrepancy also affects the interpretation of LOQ and LOD for each assay, so future studies will need to determine their own LOD and LOQ, which is standard practice. As with any qPCR study, careful attention should be paid to the choice of standards to ensure they are aligned with the goals of the study.

The challenge of developing sensitivity and specific qPCR assays is common among a number of fields including that of microbial source tracking, which aims to identify sources of microbial pollutants in the environment using molecular DNA markers, or fragments of DNA unique to a particular organism that indicate its presence. To deal with imperfect qPCR assay sensitivity and specificity, researchers in that field have used Bayesian statistics to interpret qPCR data and provide detection probabilities [46,47]. It has been demonstrated that Bayesian statistics can be useful in interpreting eDNA data using model datasets and theoretical frameworks [7,48]. With the assays presented here, given the less than ideal specificities for the humpback whale and shortbelly rockfish assays, using multiple markers or incorporating other prior knowledge such as visual data (e.g., humpback whale sightings) using a Bayesian approach could improve the interpretation of these assays when applied to field samples. Other eDNA studies have used similar approaches in detection of schistosomiasis [49], carp [50] and in assessing the impact of water filtration and PCR amplification from water samples in an aquarium [51].

Biomonitoring for common murre, rockfish, and whales in the CCE is usually carried out using visual surveys. The assays we developed here, along with assays that have been previously developed for krill, sardines, anchovies, and mackerel [37,52] could be used together to conduct biomonitoring of important vertebrate and invertebrate species from diverse trophic levels in the CCE. The murre assay is sensitive and specific and thus reliable to detect the presence of murre eDNA in a water sample. The shortbelly rockfish assay is genus-specific, meaning that positive detections indicate the presence of shortbelly rockfish, but the detection could also be from squarespot rockfish, stripetail rockfish or halfbanded rockfish. Finally, positive detections from water samples using our humpback whale assay could indicate the presence of humpback, minke, or grey whale. Because the geographic ranges of these species overlap, it will not be possible to determine which whale species is detected based on the sampling location. These assays are still useful if any other metadata or identification method is used in conjunction with the qPCR assay or if further testing is performed such as Sanger sequencing the qPCR product to confirm the identification of the target DNA.

Biomonitoring using eDNA methods provides a technique to census marine life with advantages over traditional methods including the ease of sampling, no requirement for visual identification, and longer spatial and temporal resolution of a positive detection. The extended persistence of eDNA in water sample (on the order of days) [53] enables researchers to determine where taxa have been recently, even if they are not observed visually in the exact location. The assays developed herein provide new methods to census common murre, rockfish, and whales; the assays are species-, genus-, and whale-specific, respectively. While the humpback whale assay is not species or genus-specific, we note that whale monitoring efforts heavily rely on visual observations, which are conducted infrequently and in a limited spatial scale. Presence of whales inferred from eDNA analyses, can direct future visual observation efforts, saving time, money, and effort, even if the assay is not species-specific.

## Supporting information

S1 Table Accession numbers used to design primers for each target species. (XLSX)

S2 Table Accession numbers of non-target organism used for specificity testing *in silico*. (XLSX)

S3 Table Total number of publicly available sequences for organisms that are of the same species, genus, and order of target organisms and the average number of mismatches in the forward primer, reverse primer, and probe. All of the publicly available sequences in the NBCI nucleotide database that had 5 or fewer mismatches to the forward primer, reverse primer, and/or probe were compiled for the species, genus, and order for each target organism. For humpback whale, the infraorder (Cetacea) was used rather than the order (Artiodactyla) due to the excessively large number of sequences.
(XLSX)

S4 Table Metadata of tissue samples tested for sensitivity and specificity. (XLSX)

## References

[1] Thomsen PF, Willerslev E. Environmental DNA: An emerging tool in conservation for monitoring past and present biodiversity. Biological Conservation. Elsevier Ltd; 2015;183: 4–18. 10.1016/j.biocon.2014.11.019

[2] Barnes MA, Turner CR. The ecology of environmental DNA and implications for conservation genetics. Conservation Genetics. Springer Netherlands; 2015;17: 1–17. 10.1007/s10592-015-0775-4

[3] Edgar GJ, Barrett NS, Morton AJ. Biases associated with the use of underwater visual census techniques to quantify the density and size-structure of fish populations. Journal of Experimental Marine Biology and Ecology. 2004;308: 269–290. 10.1016/j.jembe.2004.03.004

[4] Bohmann K, Evans AR, Gilbert MTP, Carvalho GR, Creer S, Knapp M, et al Environmental DNA for wildlife biology and biodiversity monitoring. Trends in Ecology & Evolution. Elsevier Ltd; 2014;29: 358–367. 10.1016/j.tree.2014.04.003 24821515

[5] Goldberg CS, Turner CR, Deiner K, Klymus KE, Thomsen PF, Murphy MA, et al Critical considerations for the application of environmental DNA methods to detect aquatic species. Methods Ecol Evol. 2nd ed. 2016;7: 1299–1307. 10.1111/2041-210X.12595

[6] Lacoursière-Roussel A, Rosabal M, Bernatchez L. Estimating fish abundance and biomass from eDNA concentrations: Variability among capture methods and environmental conditions. Mol Ecol Resour. 2016;16: 1401–1414. 10.1111/1755-0998.12522 26946353

[7] Shelton AO, O'Donnell JL, Samhouri JF, Lowell NC, Williams GD, Kelly RP. A framework for inferring biological communities from environmental DNA. 2016; 1–15. 10.1890/15-1733.1 27755698

[8] Stoeckle MY, Soboleva L, Charlop-Powers Z. Aquatic environmental DNA detects seasonal fish abundance and habitat preference in an urban estuary. Doi H, editor. PLoS ONE. 2017;12: e0175186–15. 10.1371/journal.pone.0175186 28403183 PMC5389620

[9] Kelly R, Shelton AO, Gallego R. Understanding PCR Processes to Draw Meaningful Conclusions from Environmental DNA Studies. Sci Rep. Springer US; 2019;9: 1–14. 10.1038/s41598-018-37186-2 31431641 PMC6702206

[10] Andruszkiewicz EA, Starks HA, Chavez FP, Sassoubre LM, Block BA, Boehm AB. Biomonitoring of marine vertebrates in Monterey Bay using eDNA metabarcoding. PLoS ONE. 2017;12: e0176343–20. 10.1371/journal.pone.0176343 28441466 PMC5404852

[11] Furlan EM, Gleeson D, Hardy CM, Duncan RP. A framework for estimating the sensitivity of eDNA surveys. Mol Ecol Resour. 2015;16: 641–654. 10.1111/1755-0998.12483 26536842

[12] Thomsen PF, Moller PR, Sigsgaard EE, Knudsen SW, Jorgensen OA, Willerslev E. Environmental DNA from seawater samples correlate with trawl catches of subarctic, deepwater fishes. PLoS ONE. 2016;11: e0165252–22. 10.1371/journal.pone.0165252 27851757 PMC5112899

[13] Fautin D, Dalton P, Incze LS, Leong J-AC, Pautzke C, Rosenberg A, et al An Overview of Marine Biodiversity in United States Waters. Ropert-Coudert Y, editor. PLoS ONE. Public Library of Science; 2010;5: e11914–47. 10.1371/journal.pone.0011914 20689852 PMC2914028

[14] Huyer A. Coastal upwelling in the California current system. Progress in Oceanography. 1983;12: 259–284. 10.1016/0079-6611(83)90010-1

[15] Santora JA, Field JC, Schroeder ID, Sakuma KM, Wells BK, Sydeman WJ. Spatial ecology of krill, micronekton and top predators in the central California Current: Implications for defining ecologically important areas. Progress in Oceanography. Elsevier Ltd; 2012;106: 154–174. 10.1016/j.pocean.2012.08.005

[16] Witteveen B, Worthy G, Wynne K, Hirons A, Andrews A III, Markel R. Trophic Levels of North Pacific Humpback Whales (Megaptera novaeangliae) Through Analysis of Stable Isotopes: Implications on Prey and Resource Quality. Aquat Mamm. 2011;37: 101–110. 10.1578/AM.37.2.2011.101

[17] Bolin RL, Abbot DP. Studies on the marine climate and phytoplankton of the central coastal area of California, 1954–1960. California Cooperative Oceanic Fisheries Investigations Reports. 1962;9: 1–23.

[18] Barlow J, Forney K. Abundance and population density of cetaceans in the California Current ecosystem. Fishery Bulletin. 2007;105: 509–526.

[19] Tonachella N, Nastasi A, Kaufman G, Maldini D, Rankin RW. Predicting trends in humpback whale (Megaptera novaeangliae) abundance using citizen science. Pac Conserv Biol. 2012;18: 297–14. 10.1071/PC120297

[20] Sakuma KM, Bjorkstedt EP, Ralston S. Distribution of pelagic juvenile rockfish (*Sebastes* spp.) in relation to temperature and fronts off Central California. California Cooperative Oceanic Fisheries Investigations Reports. 2013;54: 167–179.

[21] Keller A, Wallace J, Horness B, Hamel O, Stewart I. Variations in eastern North Pacific demersal fish biomass based on the U.S. west coast groundfish bottom trawl survey (2003–2010). Fishery Bulletin. 2012;110: 205–222.

[22] Edwards M, Beaugrand G, Hays G, Koslow JA, Richardson A. Multi-decadal oceanic ecological datasets and their application in marine policy and management. Trends in Ecology & Evolution. Elsevier Ltd; 2010;25: 602–610. 10.1016/j.tree.2010.07.007 20813425

[23] Kendall A. An Historical Review of *Sebastes* Taxonomy and Systematics. Marine Fisheries Review. 2000;62: 1–23.

[24] Krafft BA, Krag LA, Engås A, Nordrum S, Bruheim I, Herrmann B. Quantifying the Escape Mortality of Trawl Caught Antarctic Krill (Euphausia superba). Ban S, editor. PLoS ONE. 2016;11: e0162311–19. 10.1371/journal.pone.0162311 27622510 PMC5021277

[25] Croll DA. Auks At Sea. Studies in Avian Biology. 1990;14: 139–148.

[26] Mills K, Laidig T, Ralston S, Sydeman W. Diets of top predators indicate pelagic juvenile rockfish (Sebastes spp.) abundance in the California Current System. Fisheries Oceanography. 2007;16: 273–283. 10.1111/j.1365-2419.2006.00429.x

[27] Boehm AB, Grant SB. A steady state model of particulate organic carbon flux below the mixed layer and application to the Joint Global Ocean Flux Study. J Geophys Res. 2001;106: 227–231.

[28] Jackson JA, Baker CS, Vant M, Steel DJ, Medrano-Gonzalez L, Palumbi SR. Big and Slow: Phylogenetic Estimates of Molecular Evolution in Baleen Whales (Suborder Mysticeti). Molecular Biology and Evolution. 2009;26: 2427–2440. 10.1093/molbev/msp169 19648466

[29] Baker CS, Steel D, Calambokidis J, Falcone E, González-Peral U, Barlow J, et al Strong maternal fidelity and natal philopatry shape genetic structure in North Pacific humpback whales. Mar Ecol Prog Ser. 2013;494: 291–306. 10.3354/meps10508

[30] Ye J, Coulouris G, Zaretskaya I, Cutcutache I, Rozen S, Madden TL. Primer-BLAST: A tool to design target-specific primers for polymerase chain reaction. BMC Bioinformatics. 2012;13: 134 10.1186/1471-2105-13-134 22708584 PMC3412702

[31] Sievers F, Wilm A, Dineen D, Gibson TJ, Karplus K, Li W, et al Fast, scalable generation of high-quality protein multiple sequence alignments using Clustal Omega. Molecular Systems Biology. Nature Publishing Group; 2011;7: 1–6. 10.1038/msb.2011.75 21988835 PMC3261699

[32] Goujon M, McWilliam H, Li W, Valentin F, Squizzato S, Paern J, et al A new bioinformatics analysis tools framework at EMBL-EBI. Nucleic Acids Research. 2010;38: W695–W699. 10.1093/nar/gkq313 20439314 PMC2896090

[33] McWilliam H, Li W, Uludag M, Squizzato S, Park YM, Buso N, et al Analysis Tool Web Services from the EMBL-EBI. Nucleic Acids Research. 2013;41: W597–W600. 10.1093/nar/gkt376 23671338 PMC3692137

[34] Wagih O. ggseqlogo: A versatile R package for drawing sequence logos. Hancock J, editor. Bioinformatics. 2017;33: 3645–3647. 10.1093/bioinformatics/btx469 29036507

[35] Schneider TD, Stephens RM. Sequence logos: a new way to display consensus sequences. Nucleic Acids Research. 1990;18: 6097–6100. 10.1093/nar/18.20.6097 2172928 PMC332411

[36] Miller S, Dykes D, Polesky H. A simple salting out procedure for extracting DNA from human nucleated cells. Nucleic Acids Research. 1988;16: 1215 10.1093/nar/16.3.1215 3344216 PMC334765

[37] Yamahara KM, Preston C, Birch J, Walz K, Marin R III, Jensen S, et al In situ Autonomous Acquisition and Preservation of Marine Environmental DNA Using an Autonomous Underwater Vehicle. Front Mar Sci. 2019; 1–14. 10.3389/fmars.2019.00373

[38] Djurhuus A, Port J, Closek CJ, Yamahara KM, Romero-Maraccini O, Walz KR, et al Evaluation of Filtration and DNA Extraction Methods for Environmental DNA Biodiversity Assessments across Multiple Trophic Levels. Front Mar Sci. 2017;4: 403–11. 10.3389/fmars.2017.00314

[39] Tynan CT, Ainley DG, Barth JA, Cowles TJ, Pierce SD, Spear LB. Cetacean distributions relative to ocean processes in the northern California Current System. Deep Sea Research Part II: Topical Studies in Oceanography. 2005;52: 145–167. 10.1016/j.dsr2.2004.09.024

[40] Hyde JR, Vetter RD. The origin, evolution, and diversification of rockfishes of the genus Sebastes (Cuvier). Molecular Phylogenetics and Evolution. 2007;44: 790–811. 10.1016/j.ympev.2006.12.026 17320419

[41] Bayha KM, Collins AG, Gaffney PM. Multigene phylogeny of the scyphozoan jellyfish family Pelagiidae reveals that the common U.S. Atlantic sea nettle comprises two distinct species (*Chrysaora quinquecirrha* and *C*. *chesapeakei*). PeerJ. 4 ed. 2017;5: e3863–43. 10.7717/peerj.3863 29043109 PMC5642265

[42] Alberts B, Bray D, Lewis J, Raff M, Roberts K, Watson J. Molecular Biology of the Cell. W. W. Norton & Company; 2014.

[43] Quiros PM, Goyal A, Jha P, Auwerx J. Analysis of mtDNA/nDNA Ratio in Mice. Auwerx J, Ackerman SL, Brown SD, Justice MJ, Nadeau J, editors. Current Protocols in Mouse Biology. 2017;7: 47–54. 10.1002/cpmo.21 28252199 PMC5335900

[44] Guo W, Jiang L, Bhasin S, Khan SM, Swerdlow RH. DNA extraction procedures meaningfully influence qPCR-based mtDNA copy number determination. Mitochondrion. 2009;9: 261–265. 10.1016/j.mito.2009.03.003 19324101 PMC2798162

[45] Moushomi R, Wilgar G, Carvalho G, Creer S, Seymour M. Environmental DNA size sorting and degradation experiment indicates the state of Daphnia magna mitochondrial and nuclear eDNA is subcellular. Sci Rep. Springer US; 2019;9: 1–9. 10.1038/s41598-018-37186-2 31467341 PMC6715800

[46] Kildare BJ, Leutenegger CM, McSwain BS, Bambic DG, Rajal VB, Wuertz S. 16S rRNA-based assays for quantitative detection of universal, human-, cow-, and dog-specific fecal Bacteroidales: A Bayesian approach. Water Research. 2007;41: 3701–3715. 10.1016/j.watres.2007.06.037 17644149

[47] Curtis K, Gonzalez RA. Integrating Bayesian Analysis and Cumulative Probability Generates High Confidence Using a Single Microbial Source Tracking Marker. Environ Sci Technol. American Chemical Society; 2019;53: 13929–13937. 10.1021/acs.est.9b03843 31682415

[48] Lahoz-Monfort JJ, Guillera-Arroita G, Tingley R. Statistical approaches to account for false-positive errors in environmental DNA samples. Mol Ecol Resour. 2015;16: 673–685. 10.1111/1755-0998.12486 26558345

[49] Sengupta ME, Hellstrom M, Kariuki H, Olsen A, Thomsen P, Mejer H, et al Environmental DNA for improved detection and environmental surveillance of schistosomiasis. PNAS. 2019;116: 8931–8940. 10.1073/pnas.1815046116 30975758 PMC6500138

[50] Schultz MT. Inference of genetic marker concentrations from field surveys to detect environmental DNA using Bayesian updating. 2018; 1–20. 10.1371/journal.pone.0190603 29381694 PMC5790220

[51] Doi H, Fukaya K, Oka S-I, Sato K, Kondoh M, Miya M. Evaluation of detection probabilities at the water-filtering and initial PCR steps in environmental DNA metabarcoding using a multispecies site occupancy model. Sci Rep. Springer US; 2019; 1–8. 10.1038/s41598-018-37186-2 30837589 PMC6401178

[52] Sassoubre LM, Yamahara KM, Gardner LD, Block BA, Boehm AB. Quantification of environmental DNA (eDNA) shedding and decay rates for three marine fish. Environ Sci Technol. 2016;50: 10456–10464. 10.1021/acs.est.6b03114 27580258

[53] Collins RA, Wangensteen OS, O’Gorman EJ, Mariani S, Sims DW, Genner MJ. Persistence of environmental DNA in marine systems. Communications Biology. Springer US; 2018; 1–11. 10.1038/s42003-018-0192-6 30417122 PMC6218555

